# Findings of prosthetic valve endocarditis in the balloon-expandable trans-catheter aortic valve: review of the literature and tips of management

**DOI:** 10.1186/s13019-021-01609-5

**Published:** 2021-08-16

**Authors:** Domenico Calcaterra, Kevin Harris, Mario Goessl, Gopika Dasari, Navneet Kaur, Ivan Chavez

**Affiliations:** 1 Florida Atlantic University, 777 Glades Road, Boca Raton, FL 33431 USA; 2 Minneapolis Heart Institute Foundation, Minneapolis, MN USA

**Keywords:** TAVI, TAVI-endocarditis, TAVI explant, Posterior aortic root enlargement

## Abstract

Prosthetic valve endocarditis after transcatheter aortic valve implantation (TAVI) is a rare complication associated with a high mortality rate. Nonetheless, the rapid expansion of TAVI in recent years has proportionally increased the number of patients exposed to the risk of developing transcatheter valve infection. A 71-year-old female with recent history of TAVI was diagnosed with prosthetic valve obstruction secondary to endocarditis. The characteristics of clinical presentation of endocarditis in the balloon-expandable transcatheter valve and the intra-operative findings are discussed with a review of the literature and tips of management.

## Case presentation

**Fig. 1:**
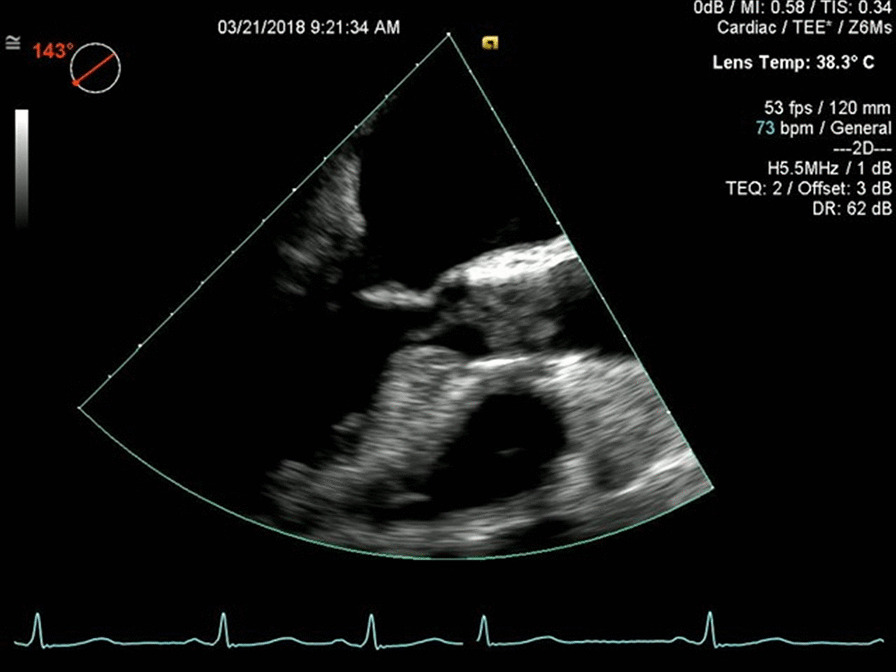
Preoperative trans-esophageal echocardiographic image showing obstruction to the valve orifice caused by the intraluminal growth of the infectious vegetation

**Fig. 2:**
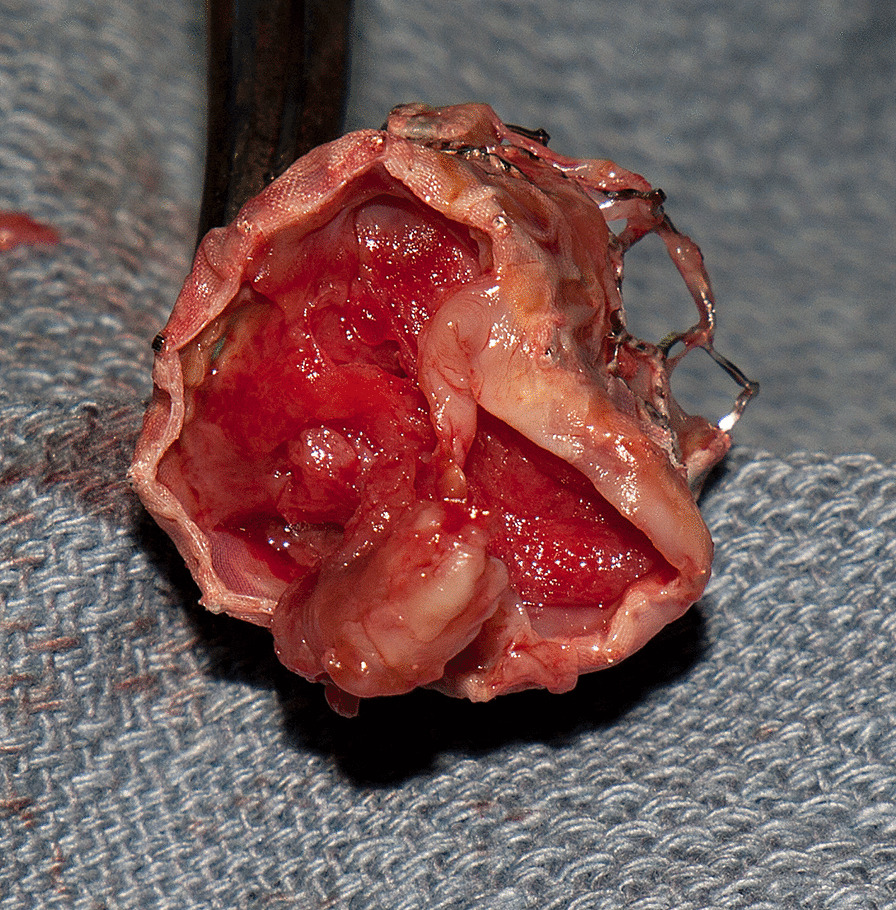
Surgical specimen with bottom view of the balloon-expandable trans-catheter valve prosthesis showing near-complete obstruction of the prosthesis orifice valve area by the endoluminal growth of the infectious vegetation

**Fig. 3:**
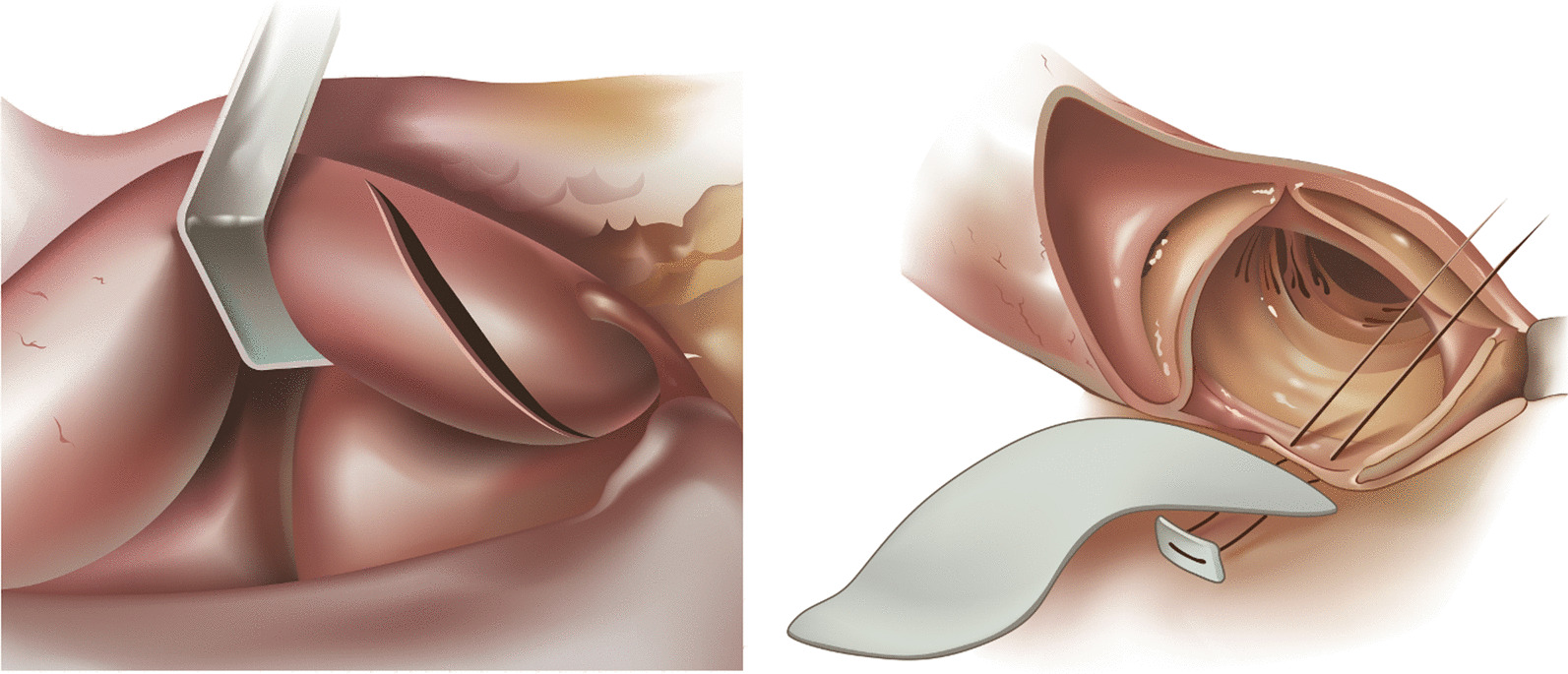
Technique of posterior aortic root enlargement

A 71-year-old female presented with lethargy, confusion, dysarthria, and congestive heart failure New York Heart Association (NYHA) functional class 2–3, 4 months after a transcatheter aortic valve implantation. She had undergone implantation of a 23 mm SAPIEN 3 (Edwards Lifesciences Corporation, Irvine, CA USA) as a high to intermediate risk surgical replacement candidate, with access through the right axillary artery. The patient was affected by morbid obesity with a BMI of 54 and had inadequate femoral arterial access. At time of re-admission to the hospital, she was found to have a low-grade fever in absence of leukocytosis and a grade 3/6 systolic ejection murmur. Head computed tomography scan (CT scan) showed multiple left hemisphere parietal and occipital acute embolic infarcts, with no evidence of hemorrhagic conversion. Transesophageal echocardiogram (TEE) revealed a large mobile vegetation (0.4 × 1.5 cm) attached to the prosthetic valve leaflets associated with significant valve obstruction without regurgitation (mean gradient of 29 mmHg, peak gradient of 47 mmHg, orifice valve area of 1.7 cm^2^) (Fig. 1). The patient had a preserved ejection fraction (EF) of 60%, with left ventricular end diastolic dimension (LVEDD) of 4.8 cm. Blood cultures were positive for Streptococcus Viridans. She was started on antibiotic therapy with intravenous (IV) ceftriaxone (2 g twice a day), and pharmacologic treatment of the heart failure symptoms. Based on the existing surgical indications of distal embolization, large vegetation (> 1 cm), aortic valve obstruction with heart failure, and persistent positive blood cultures despite IV antibiotic therapy [1], once her general conditions had improved with a full neurologic recovery, she was taken to surgery 2 weeks after admission for removal of the transcatheter valve and surgical aortic valve replacement (SAVR). She underwent SAVR with a 23 mm bio-prosthesis. Intraoperatively, the trans-catheter valve prosthesis was involved by growth of a bulky vegetation obstructing the orifice valve area (Fig. 2). The small size of the aortic annulus required a posterior aortic root enlargement to facilitate maneuvers of extraction of the balloon expandable transcatheter valve and allow to accommodate an adequate size aortic valve bio-prosthesis (23 mm Mosaic Ultra™. Medtronic Inc., Minneapolis MN, USA). The posterior root enlargement was obtained according to the Manouguian technique, extending the anterior transverse aortotomy posteriorly and across the aortic valve annulus through the commissure between the left and the non-coronary sinuses. The extended aortotomy was reconstructed using a bovine pericardial patch sutured from the bottom corner to the two sides of the incision using a 4-0 polypropylene suture in a continuous fashion (Fig. 3). Pathology exam of the specimen revealed a fibrinopurulent composition of the valve vegetation, which grew Streptococcus Viridans. The operation required 144 min of cardiopulmonary bypass with 116 min of aortic cross-clamping. Cardiac arrest was obtained with infusion of cold blood cardioplegia delivered in the retrograde fashion at induction and intermittently in antegrade and retrograde fashion after aortic cross clamping. Mechanical ventilation was weaned off within 10 h and she was transferred to telemetry floor on the second postoperative day. The patient was discharged to a rehabilitation facility on the 7th postoperative day following an uncomplicated recovery and she completed a 4-week cycle of IV antibiotic therapy with ceftriaxone from the day of surgery. At a 24-month follow-up she is well with trans-thoracic echocardiography (TTE) showing EF of 60%, orifice valve area of 2.6 cm^2^ and a mean transvalvular gradient of 12 mmHg. The patient provided written consent to the publication of this report.

## Discussion

The rate of early infective endocarditis following TAVI is between 1 and 3% with a median time of diagnosis of 5 months after the initial procedure [2–4]. This condition is associated with a very high one-year in-hospital mortality of approximately 35% and a 2-year mortality rate of 67% [2, 3]. Limited data exists on the characteristics of clinical presentation. The Infectious Endocarditis after TAVI International Registry retrospectively collected data from patients diagnosed with infective endocarditis after TAVR from 47 sites across Europe, North America, and South America between 2005 and 2015 [2]. Out of a total of 20,006 cases of TAVR, 250 were diagnosed with early (within 12 months from implantation) TAVI-PVE. Of these, only 10% of the patients underwent surgical intervention with a perioperative mortality of 30%. The most common presenting symptoms were fever (80%) and heart failure symptoms (30%). Enterococcus species was the most common causative organism (25%), followed by Staphylococcus Aureus (22%) and coagulase-negative Staphylococcus (17%). The study showed that younger age, male sex, history of diabetes mellitus, and moderate to severe residual aortic regurgitation after TAVI were associated with an increased risk of developing PVE, whereas the presence of heart failure symptoms, kidney injury, and higher logistic EuroSCORE were associated with increased in-hospital mortality and late death (2). In a literature review of 28 publications of PVE following TAVR from 2000 to 2013, only 23% of patients underwent surgical intervention, with a perioperative mortality of 31%. Interestingly, the intervention rate was much higher for balloon-expandable devices (57%) when compared to self-expandable ones (23%) [3]. The recent publication of the analysis of PVE occurrence in the 8530 patients of the PARTNER-1 and PARTNER-2 trial-registries, showed that incidence of PVE between TAVR and surgical aortic valve replacement (SAVR) was comparable [4]. Predictors of occurrence of PVE in both groups were renal, lung, and liver disease. Staphylococcus was the organism more commonly responsible in SAVR-PVE, while Streptococcus was the most common cause in TAVR-PVE. Other studies demonstrated a very high morbidity and mortality of TAVR-PVE, with rates of in-hospital mortality nearly double to those seen in surgical PVE, and with a much lower rate of aortic valve reintervention, as low as 11% [4–7].

**Fig. 4:**
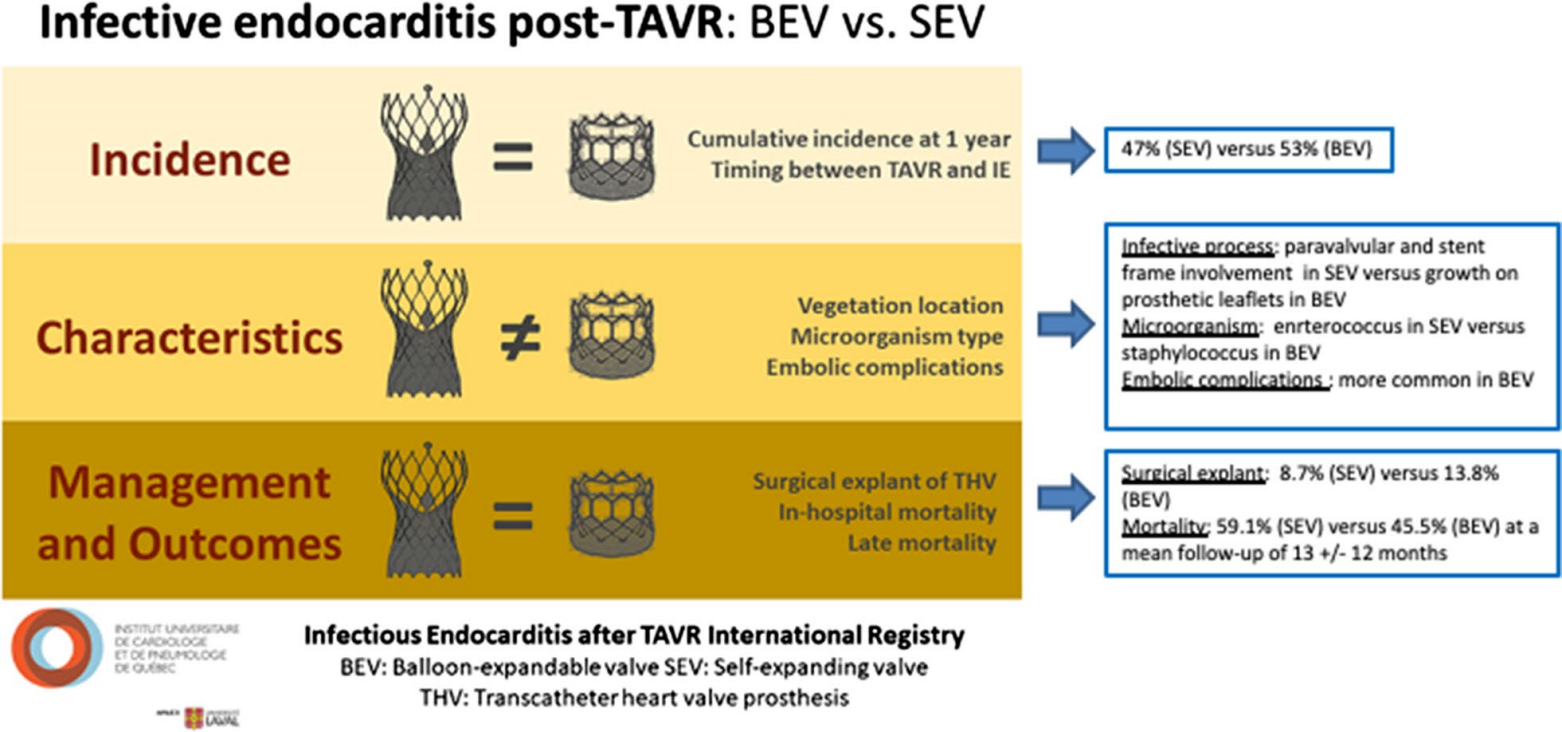
Endocarditis post-TAVR: balloon-expandable versus self-expandable valve (modified with permission from Regueiro, et al. Circ Cardiovasc Interv. 2019 Nov;12:e007938) [8]

There is one key factor regarding the pathophysiology of TAVR-PVE which differentiate the characteristics of the infectious process in the self-expandable versus the balloon-expandable valve prosthesis as shown by echocardiography findings [2, 3, 7]. In cases of endocarditis of the self-expandable valve, the vegetation growth more commonly involves the stent frame rather than the prosthetic leaflets, whereas vegetations are more commonly identified on the valve-leaflets with endocarditis of the balloon-expandable transcatheter prosthesis [2, 3, 7–9]. So, even though the overall rate of infection does not differ between the two types of device, there is a higher rate of paravalvular and aortic wall involvement in the self-expandable valve since its structure seems to favor the infectious process to preferentially develop involving the stent frame of the prosthetic valve compared to the leaflets (Fig. 4) [2–4, 6, 7, 10]. Therefore, the longer stent frame of the self-expandable valve adherent to the aortic wall, and the higher incidence of paravalvular abscess and aortic fistula, may discourage surgical intervention and may explain the lower rate of explant seen in this subgroup [2–4, 7]. In the case presented, early intervention was the key to a positive outcome and a posterior aortic root enlargement allowed for a safe explant of the transcatheter prosthesis making possible to accommodate a larger size bioprosthetic valve.

## Conclusions

TAVI-PVE is a condition associated with a dismal prognosis [2–8], in which the high surgical risk of the patients’ population and the high level of technical difficulty of the operation is often a deterrent to proceed with surgical intervention in favor of palliative medical treatment. Nonetheless, surgical aortic valve reintervention offers the best chance for cure [5, 8]. In the case presented, echocardiography and intraoperative findings show the peculiar characteristics of PVE in the balloon-expandable transcatheter valve where the infectious process preferentially involves the prosthetic leaflets, exposing to high occurrence of flow obstruction and distal embolization, with lesser risk of paravalvular complications as compared to PVE as seen in the self-expandable transcatheter valve.

## Data Availability

Not applicable.

## Abbreviations

NYHA: New York Heart Association
TAVI: Transcatheter aortic valve implantation
CT scan: Computed tomography scan
TEE: Trans esophageal echocardiography
EF: Ejection fraction
LVEED: Left ventricular end diastolic dimension
IV: Intravenous
SAVR: Surgical aortic valve replacement
TTE: Transthoracic echocardiography
PVE: Prosthetic valve endocarditis
